# Fast "coalescent" simulation

**DOI:** 10.1186/1471-2156-7-16

**Published:** 2006-03-15

**Authors:** Paul Marjoram, Jeff D Wall

**Affiliations:** 1 Department of Preventive Medicine, University of Southern California, Los Angeles, CA 90089-9011, USA; 2 Molecular and Computational Biology, University of Southern California, Los Angeles, CA 90089, USA

## Abstract

**Background:**

The amount of genome-wide molecular data is increasing rapidly, as is interest in developing methods appropriate for such data. There is a consequent increasing need for methods that are able to efficiently simulate such data. In this paper we implement the sequentially Markovian coalescent algorithm described by McVean and Cardin and present a further modification to that algorithm which slightly improves the closeness of the approximation to the full coalescent model. The algorithm ignores a class of recombination events known to affect the behavior of the genealogy of the sample, but which do not appear to affect the behavior of generated samples to any substantial degree.

**Results:**

We show that our software is able to simulate large chromosomal regions, such as those appropriate in a consideration of genome-wide data, in a way that is several orders of magnitude faster than existing coalescent algorithms.

**Conclusion:**

This algorithm provides a useful resource for those needing to simulate large quantities of data for chromosomal-length regions using an approach that is much more efficient than traditional coalescent models.

## Background

Given the increasing prevalence of genome-wide data, and the development of methodologies for the analysis of such data, there is an increasing need for tools that can simulate data appropriate for long, genomic regions. Two options suggest themselves:

1. **Model and simulation: **The traditional approach has been to use a model that is

(a) thought to be a reasonable approximation to the evolutionary history for the organism of interest, and

(b) easy to simulate.

By far the most popular such model is the *coalescent *[1,2] However, use of the coalescent becomes less practical for long genomic regions.

2. **Existing data and perturbation: **An alternate, newer approach is to take an existing data set and then perturb it in some fashion to produce "new data from old". A simple example of such an approach would be re-sampling. More specific examples can be found in [3,4].

The first approach has the advantage of being able to produce data that is not dependent on an existing data set. However, the model it uses will be, by definition, an approximation to the evolutionary processes that produced the real data. The second approach, while being dependent on the presence of an initial data set, has the advantage that the evolutionary model underlying the unperturbed data is correct. We don't know how the data got there, but it is 'real' data, so it got there via the correct evolutionary history. However, the need to then perturb the initial data to produce new data sets adds noise to the evolutionary process and thereby results in data that is only an approximation to reality. Furthermore, the extent of the dependence of the new data sets on the initial data set is unclear, and it is therefore not obvious how typical such data might be of other, unobserved, real data.

We believe both of these approaches have merit. In this paper we restrict ourselves to a discussion of the former approach, in which we use an evolutionary model to simulate new data sets. The use of the standard coalescent model becomes impractical as the length of the simulated region increases. However, the coalescent has been proven to be a powerful simulation tool in these contexts (e.g., [5]). Thus, in this paper we exploit an approximation to the full coalescent algorithm. This approximation, the sequentially Markov coalescent (SMC), was introduced by McVean and Cardin [6]. It is able to simulate significantly longer regions while maintaining the properties of short-range summary statistics. Since our particular interest is in the development of such algorithms as a tool for the testing of disease mapping methodologies, we pay close attention to the behavior of linkage disequilibrium [LD] in data simulated under the SMC model. The coalescent was introduceid in [1]. It provided an elegant and efficient model for the evolution of a population of randomly-mating, neutral, haploid individuals. As such it has become a very widely used tool. Over time, generalizations have been introduced to deal with the more obviously restrictive aspects of the original model. For example, recombination was introduced in [2]. Selection was introduced in [7,8]. Useful reviews are found in [9-11].

Our interest here centers on the use of the coalescent algorithm to simulate long chromosomal regions. When long regions are considered, and the recombination rate is therefore very high, the coalescent algorithm becomes somewhat problematic to use. Run-times become longer (see "Results") and memory requirements become greater.

In a case in which two widely-separated regions were being considered, one might simulate these two regions independently, relying on the fact that the regions would be essentially unlinked. However, when one is studying a long, continuous region such a strategy becomes inappropriate since linkage disequilibrium is likely to be present along the entire region. (In a situation in which recombination hotspots were present, one might try to independently simulate regions between hotspots.)

Rapid simulation of coalescent ancestries is central to estimation methods such as rejection algorithms, or to the use of simulation-studies as a test-bed for new methodologies. Thus we use a simple approximation to the coalescent in which the difficulties associated with simulating long chromosomal regions are mitigated.

Hudson [2] introduced recombination into the coalescent model. Griffiths and Marjoram [12] then embedded this within the ancestral recombination graph (ARG), a more tractable description of the coalescent model in the presence of recombination. Shortly thereafter, Wiuf and Hein [13,14] introduced an alternate description of the ancestral process with recombination in which the sample is constructed by moving "along the chromosome". Their algorithm gains efficiency by ignoring a class of recombination events that do not affect the present day sample.

In order to discuss this further, we review the concept of *ancestral *material. A chromosomal region in an individual is considered to be ancestral if it is eventually inherited by any of the sample of interest drawn from the present day population. Thus, individuals in previous generations are likely to contain chromosomal regions that are both ancestral and non-ancestral.

In essence there are five types of recombination events that occur on the full ARG:

1. Recombination in ancestral material;

2. Recombination in non-ancestral material that has ancestral material to both sides;

3. Recombination in non-ancestral material that has ancestral material only to the left;

4. Recombination in non-ancestral material that has ancestral material only to the right;

5. Recombination in an individual that carries no ancestral material.

**Figure 1:**
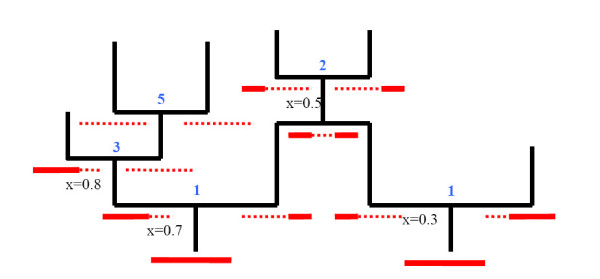
**The various categories of recombination**. Illustration of the different types of recombinations. Ancestral material is shown as solid red lines, while non-ancestral material is shown as red-dotted lines. Locations of recombinations are shown below and to the left of the recombination event. Type of recombination is indicated with a blue numeral above the event.

We illustrate some of these events in Figure 1 [see Additional file 1]. Only the first two types of event actually impact the composition of the sample of interest.

As the recombination parameter, *ρ*, increases, the number of recombinations in the ARG, which is of the order of *ρ*log(*n*) for a sample of size *n*, (e.g., [12]) grows. A simulation of the full ARG would contain all such recombination events, and hence be highly inefficient. This is not, primarily, due to the large number of recombination events *per se*, but rather is caused by the growing size of the ARG, which makes increasing demands on computer memory.

### Simulating the ancestral recombination graph

**Figure 2:**
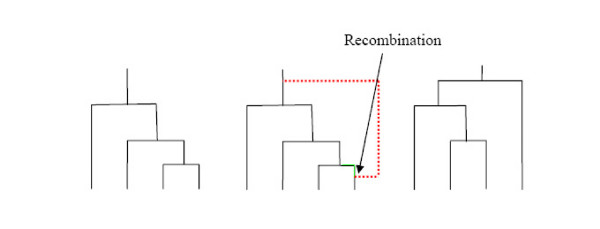
**Illustration of *FastCoal *algorithm**. This figure shows how the algorithm forms the next tree along the chromosome, moving from left-to-right, given the state of the current tree.

We begin by introducing some notation. Denote the length of chromosome being considered by the unit interval [0,1]. Let *x *∈ [0,1] denote a point within the region of interest. McVean and Cardin's SMC method introduces an approximation to an elegant scheme introduced by Wiuf and Hein [13,14], which we describe fully in "Implementation". In summary, Wiuf and Hein's method moves from left-to-right along the chromosome. Starting with the tree appropriate for *x *= 0 they find the (exponentially distributed) distance along the chromosome to the next recombination event. They then pick a point uniformly at random on the graph constructed so far and introduce a recombination at that point. The left emerging line from that recombination follows the path of the existing line (indicated in green on Figure 2 – [see Additional file 2]), but the right emrging line, which is the newly-introduced line, follows a new path (calculated from the usual coalescent prior and indicated in red on Figure 2). Once we have constructed the path for the new line we are left with a new graph that consists of the old graph plus this new line. This procedure is iterated until the end of the chromosome is reached. Note that the size of the graph increases as *x *increases. (For details see "Implementation".)

There is a class of recombination events which occur to lines on the full ARG but which do not affect the properties of samples generated from that graph. These are recombinations which occur in regions which are non-ancestral for that line (i.e. are not passed on to the sample of interest at the bottom of the graph) and which do not have ancestral regions to both their left and right (corresponding to events of types 3, 4 and 5 in the list of the previous section). The Wiuf and Hein algorithm gains efficiency over an algorithm based on the full ARG by excluding recombinations of types 4 and 5. In particular, it excludes recombinations which occur in non-ancestral material and which only have ancestral material to their right on that line, but not those that have ancestral material only to their left. This has the curious feature of making the density of recombination events in the simulated graph increase as we move along the chromosomal region. (However, it is important to note that these 'extra' recombination events occur in non-ancestral material and do not influence the composition of the final sample.) Since, for large *ρ*, the efficiency of algorithms that simulate (a subset of) the ARG is largely a function of the amount of memory required to store the graph, this makes the Wiuf and Hein algorithm more efficient than algorithms based on the full ARG. The popular ms algorithm of Hudson [15] also excludes recombinations in non-ancestral material that only have ancestral material to their left (i.e. of type 3). Thus, all events that do not affect the current sample are ignored in the ms algorithm, and it is therefore more efficient than the Wiuf and Hein algorithm.

The novelty of the SMC scheme proposed by McVean and Cardin, is that before adding the new line corresponding to a recombination event they delete the old (existing) line for that recombination, (i.e. all parts of the old line between the point at which the recombination occurred, and the point at which the old line coalesced, are deleted). Thus, each graph we construct is in fact a tree, and knowledge of the deleted lines is lost rather than being stored within the total graph constructed so far (as in the algorithm of Wiuf and Hein). Consequently, the SMC algorithm explicitly disallows coalescence between lineages with no overlapping ancestral material. The motivation is to significantly increase algorithmic efficiency, due to lower memory requirements, while retaining most of the LD structure [6]. This is possible largely because the process by which trees are constructed as we move along the chromosome using the SMC is now Markovian.

In our implementation of their algorithm we include a slight modification in which the old (existing) line for the recombination is deleted *after *(rather than before) the new line is added. Thus, in this modified version, which we refer to as SMC', the intuitive interpretation is that we only include recombination events that occur in ancestral material and ignore all events occurring in non-ancestral regions. Our motivation for doing this is as follows. The original specification of the SMC algorithm has the consequence of excluding a class of recombination events that occur in ancestral material but do not affect the pattern of LD in the data (because the two lines resulting from the recombination coalesce with each other before coalescing with any other line). We denote this class of recombinations by *R*. Thus, since all recombinations are forced to be other than type *R*, the rate at which recombinations of type not equal to *R *occurs will now be slightly higher than it would normally be under the full coalescent model (for a given recombination parameter *ρ*). This suggests that LD will decay slightly more quickly in the original, SMC, version of the algorithm (see "Results" for more details). Our *FastCoal *software implements both the SMC and SMC' versions of the algorithm.

We give results to supplement those in [6] and demonstrate that the SMC/SMC' approximation is much more efficient than the coalescent for high *ρ *and produces data that is almost indistinguishable from that produced by an exact coalescent algorithm. The algorithm simulates an *approximation *to the true coalescent model. However, the degree of approximation is extremely close, at least in terms of patterns of LD. Furthermore, implementation of the algorithm results in software that is very significantly faster, and has much lower memory requirements, when *ρ *is large. In fact, the memory required by the algorithm is independent of *ρ *(in direct contrast to ms, for example).

Note that by discarding the old line associated with each recombination, at any given moment the algorithm stores information for one coalescent tree rather than a more complicated and memory-intensive graph. This allows the SMC/SMC' algorithm to run efficiently for high *ρ *(when the size of the corresponding graph becomes very large). Other than that, the SMC/SMC' process and Wiuf & Hein algorithms are essentially the same. Thus it follows, in a manner directly analogous to that in [13,14], that T(*x*), the marginal genealogy at a particular location *x*, is still exactly described by the traditional coalescent process. See [6] for an extended discussion of the properties of the SMC algorithm and a derivation of theoretical results.

We now consider the degree to which data produced by the approximation algorithm is similar to that produced by traditional coalescent algorithms and then demonstrate the relative computational efficiencies for a variety of parameter values.

## Implementation

### Wiuf and Hein's algorithm

We start by summarizing Wiuf and Hein's method [13,14]. Their algorithm provides a way of constructing a subset of the ARC by moving 'along the chromosome', constructing the tree appropriate for each point on the chromosome and storing those trees within a graph that is a subset of the full ARG. Recall that *x *denotes a location in the interval being simulated. The algorithm proceeds as follows:

1. Set *x *= 0 and generate a coalescent tree for *x*. Denote this tree by *G*(*x*). Denote the length of the graph at *x *by *L*(*x*).

2. Generate *y *~ Exp(ρ2
 MathType@MTEF@5@5@+=feaafiart1ev1aaatCvAUfKttLearuWrP9MDH5MBPbIqV92AaeXatLxBI9gBaebbnrfifHhDYfgasaacH8akY=wiFfYdH8Gipec8Eeeu0xXdbba9frFj0=OqFfea0dXdd9vqai=hGuQ8kuc9pgc9s8qqaq=dirpe0xb9q8qiLsFr0=vr0=vr0dc8meaabaqaciaacaGaaeqabaqabeGadaaakeaadaWcaaqaaGGaciab=f8aYbqaaGGaaiab+jdaYaaaaaa@2F75@*LM*(*x*)), the distance along the chromosome to the next recombination event.

3. Pick a point *g *on the graph *G*(*x*)uniformly.

4. Add a recombination event to the graph at that *g*. The recombination occurs at chromosomal location *x + y*. The left emerging branch follows the path of the existing line at that point. The right emerging line coalesces at some point higher up on the graph (possibly past the MRCA) according to the usual coalescent probabilities. In particular, it coalesces with each existing line at rate 1.

5. Set *x = x *+ *y*. Let *G*(*x*) denote the total graph constructed so far (i.e. *G*(*x*) contains all branches appropriate for any *z < x*). Set *L*(*x*) equal to the total length of *G*(*x*).

6. If *x *+ *y <*1 return to 2.

The method of Wiuf and Hein simulates a substantial sub-sample of the full ARG. Thus, its burden on computer memory is also substantial, to the point of being intractable for long genomic regions. McVean and Cardin introduced the SMC algorithm, an approximation to the process of Wiuf and Hein. The SMC algorithm reduces the topology being simulated to a tree rather than a graph. We now introduce our variation on the SMC algorithm, which we refer to as SMC'.

### The SMC' algorithm

The SMC' algorithm proceeds as follows:

1. Set *x *= 0 and generate a coalescent tree for *x*. Denote this tree by *T*(*x*). Denote the length of the tree at *x *by *L*(*x*).

2. Generate *y *~ Exp(ρ2
 MathType@MTEF@5@5@+=feaafiart1ev1aaatCvAUfKttLearuWrP9MDH5MBPbIqV92AaeXatLxBI9gBaebbnrfifHhDYfgasaacH8akY=wiFfYdH8Gipec8Eeeu0xXdbba9frFj0=OqFfea0dXdd9vqai=hGuQ8kuc9pgc9s8qqaq=dirpe0xb9q8qiLsFr0=vr0=vr0dc8meaabaqaciaacaGaaeqabaqabeGadaaakeaadaWcaaqaaGGaciab=f8aYbqaaGGaaiab+jdaYaaaaaa@2F75@*L*(*x*)), the distance along the chromosome to the next recombination event.

3. Pick a point *g *on the tree *T*(*x*) uniformly.

4. Add a recombination event to the graph at that *g*. The recombination occurs at chromosomal location *x *+ *y*. The left emerging branch follows the path of the existing line at that point. We refer to this as the old branch. The right emerging line coalesces at some point higher up on the graph (possibly past the MRCA) according to the usual coalescent probabilities. In particular, it coalesces with each existing line at rate 1.

5. Delete the part of the old (i.e. left) branch that lies between the newly added recombination event and the point at which the old branch coalesces with another line. At this point we are left with a tree (rather than a graph).

6. Set *x = x *+ *y*. Let *T*(*x*) denote the tree constructed at *x*. Set *L*(*x*) equal to the length of *T*(*x*).

7. If *x *+ *y <*1 return to 2.

We illustrate this algorithm in Figure 2 [see Additional file 2]. In the original SMC algorithm presented by McVean and Cardin steps 4 and 5 are conducted in reverse order.

As we discussed earlier, this algorithm has the property that, at any point in time, the topology being considered is a tree rather than a graph. Furthermore, as discussed in [6], the algorithm is now Markovian as we move along the chromosome. As such it can be efficiently stored in memory, the amount of memory required being independent of the recombination rate.

## Results

### Behavior of algorithm – tree heights

As noted in [6], exploiting the approximation described by the SMC algorithm (or the SMC' variation), implies that we are no longer simulating exact coalescent ancestries. For the reasons discussed above and in [6] it rather straightforwardly follows that time to most recent common ancestor (TMRCA) for any point *x *∈ [0,1] will have the same distribution as for the standard coalescent.

It is of some interest to consider the mean height of the *i*th tree moving (from left-to-right) along the chromosome. Results for ms, SMC and SMC' are shown in Table 1 for a sample of size *n *= 2 and *ρ *= 1 and 100. Note that the *i*th tree will not always exist. The mean heights presented for tree *i *in Tables 1 and 2 are conditional on the existence of the *i*th tree (for each *i*). (Thus, iterations for which the *i*th tree does not exist are not used for the calculation of the mean height of tree *i*.)There are two intuitions underlying the results shown in the table. Firstly the results illustrate a subtlety first discussed in [13,14], in that the (*i *+ l)th tree is most likely to exist if the *i*thtree has a higher TMRCA than usual. Thus, conditioning on the existence of the *i*th tree leads to an increase in the mean height of that tree. Clearly, this conditioning does not apply to the first tree, or the last tree (since both always exist) – thus their mean height is unchanged. Furthermore, the extent of this effect decreases as the recombination rate increases (since the *i*th tree becomes more likely to exist as *ρ *increases). Secondly, there is a difference in behavior between the tree at x, for some position x, and the *i*th tree along the chromosome. The former has a lower expected height than the latter, since tall trees are likely to cover a shorter length of the chromosome. For *n *= 2 this is akin to size-biased sampling of exponential random variables. A little thought reveals that for high *ρ *and small *i*, the *i*th tree will exist with very high probability, the effect of the conditioning is therefore lost, and the expected tree height will be approximately 2 due to the size-biasing effect. The effect of the size-biasing is lessened at each end of the region.

**Table 1:** Mean Height of *i*th tree for SMC. We show the mean TMRCA for the *i*th tree along the chromosome, when it exists, as a function of the recombination rate. Data was simulated for a sample size of *n *= 2. Results are given for ms, SMC and SMC'.

	*ρ *= 1			*ρ *= 100		
		
*i*	ms	SMC	SMC'	ms	SMC	SMC'
1	1.00	1.00	1.00	1.00	1.00	1.00
2	1.68	1.75	1.68	1.41	1.51	1.41
3	2.06	2.11	2.08	1.63	1.76	1.64
4	2.39	2.33	2.40	1.75	1.88	1.77
5	2.68	2.47	2.66	1.81	1.94	1.85
6	2.95	2.57	2.90	1.87	1.97	1.90
7	3.19	2.65	3.12	1.89	1.99	1.93
8	3.44	2.72	3.33	1.91	1.99	1.95
9	3.67	2.79	3.54	1.93	1.99	1.97
10	3.92	2.84	3.69	1.94	2.00	1.98

last tree	1.00	1.00	1.00	1.00	1.00	1.00

**Table 2:** Mean Height of *i*th tree for SMC. We show the mean TMRCA for the *i*th tree along the chromosome, when it exists, for a sample size of *n *= 20. Results are given for ms, SMC and SMC'

	*ρ *= 100		
	
*i*	ms	SMC	SMC'
1	1.90	1.90	1.90
2	1.96	1.99	1.96
3	1.99	2.05	2.01
4	2.05	2.10	2.05
5	2.06	2.14	2.08
6	2.09	2.16	2.11
7	2.11	2.19	2.13
8	2.12	2.20	2.15
9	2.13	2.22	2.16
10	2.15	2.22	2.18

last tree	1.90	1.90	1.90

The results show that the SMC' algorithm appears to provides a closer approximation to the full coalescent model than does SMC. As one might expect *a priori*, the degree of difference decreases as the sample size increases. We illustrate this in Table 2, where results are shown for a sample size of 20.

It is not clear how this difference in behavior might affect the properties of the data being simulated, but it suggests that the covariance of the tree heights at any two positions *x *and *y *along the region of interest will be highest under ms, with SMC' leading to a somewhat lower covariance and SMC leading to a further lowering of the covariance. (For further evidence of this effect see the results for LD below.)

### Behavior of algorithm – run times

We compare the run times of our software with those of ms (Hudson 2002). We concentrate on parameter values that are appropriate for modelling the data that will come from future large-scale association studies. All simulations assume that *θ *= 4*Nu *= 10^-4 ^(where N is the effective population size and *u *is the mutation rate per base pair per generation) and *ρ *= 4*Nr *= 5 * 10^-4 ^(where *r *is the crossover rate per base pair per generation). The *θ *value gives a SNP density of one SNP with a minor allele frequency (MAF) of at least 0.05 every 3.4 Kb and the *ρ *value assumes plausible human parameter values (N = 10^4 ^and an average recombination rate of 1.25 cM/Mb).

Table 3 shows the average time per simulation for our implementation of SMC/SMC' as a function of the sample size and length of region simulated. (Results for both versions of the algorithm are essentially identical.) For simulations of smaller regions (e.g., 200 Kb or less), the run times of SMC/SMC' and ms are roughly comparable (results not shown). However, for larger regions, the new algorithm is much faster than ms. When the simulated region is larger than a few Mb, ms could not be run due to memory constraints. We anticipate that roughly 32 GB of RAM and 2–6 days of computing time would be necessary to simulate data from a small chromosome (*n *= 4000 and 50 Mb of sequence) using the standard coalescent. In contrast, the corresponding simulations with the new algorithm take less than 2 minutes to run and use less than 200 MB of RAM. We note in passing that, as expected, the run time for our software is roughly proportional to the length of the sequence simulated. Run times for ms increase more than quadratically with respect to the simulated sequence length (results not shown).

**Table 3:** Run-times. Average time per simulation, as a function of sample size *n*, based on 20 trials, assuming *θ *= 10^-4^/bp and *ρ *= 5 * 10^-4^/bp. Simulations were run on a 2.8 GHz Intel Xeon processor. Dashes correspond to simulations that could not be completed because they required too much (> 3 GB RAM) memory.

*n*	Length (Mb)	SMC	ms
1000	2	0.9	7.2
	5	2.1	62.6
	10	4.3	473.6
	20	8.3	6459.6
	50	20.9	-
	100	41.6	-
	200	83.9	-

4000	2	4.0	10.6
	5	10.4	-
	10	22.2	-
	20	40.7	-
	50	105.8	-
	100	201.5	-
	200	406.1	-

### Behavior of algorithm – LD

**Figure 3:**
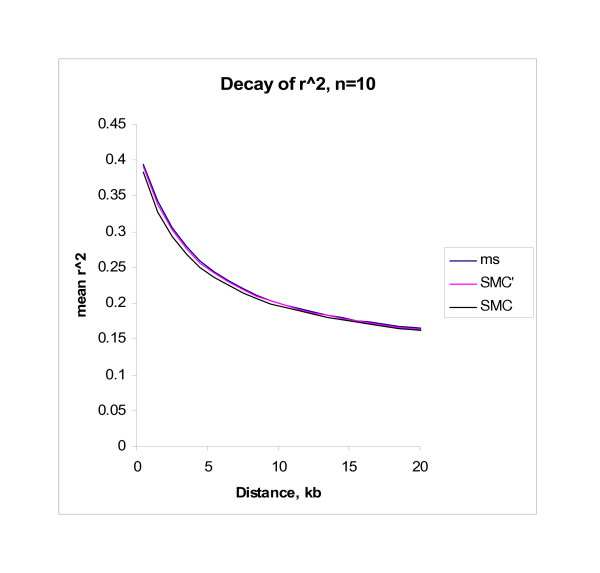
**Decay of *r*^2^**. This figure shows how *r*^2 ^decays as a function of distance for both the SMC and SMC' algorithm and for an exact coalescent model (simulated using ms). Data was simulated for a 2 Mb region and a sample size of *n *= 20.

We also compared the behavior of LD in data simulated by SMC, SMC' and ms. In Figure 3 [see Additional file 3] we simulated 2 Mb of sequence from a sample size of *n *= 10 using 1,000 replicates. As before we assumed *θ *= 10^-4^/bp, *θ *= 5 * 10^-4^/bp. In Table 4 we use *n *= 100. We illustrate the behavior of several simple summaries of LD: *r*^2 ^as a function of distance, the number of distinct haplotypes (*H*), the minimum number of inferred recombination events *R*_*M *_(cf. [16]) and the fraction of sequence contained in haplotype blocks (cf. [17]). The means of these summaries are displayed in Table 4 and Figure 3. As measured, the algorithms produce nearly identical patterns of LD, although, somewhat surprisingly SMC leads to a slightly lower value of *R*_*M*_. We note that SMC' produces a slightly closer approximation to the full coalescent model than does SMC. This is true for all sample sizes, but we note that the degree of difference between the algorithms decreases as the sample size increases, and will, for many purposes, be insignificant. We simulated a range of other parameter values (including sample sizes ranging up to 2500) and considered several other measures of LD [18,19], including patterns of LD within triplets of sites. In all cases the broad conclusions were essentially the same (results not shown). We conclude that the SMC/SMC' algorithm produces simulated data that has LD properties that are virtually indistinguishable from those resulting from standard coalescent simulations.

**Table 4:** Mean of LD summaries. We show results for the mean value of summaries of LD behavior for ms and the SMC and SMC' algorithms. We simulated a 2 Mb region for a sample of size 500. Numbers shown are averaged over 1000 replicates. Markers with MAF less than 0.05 were excluded.

Statistic	ms	SMC	SMC'
*H*	404	404	404
*R_M_*	236	232	236
% seq. in hap. blocks	41.1	41.2	41.2

## Discussion

Hudson's ms algorithm is an excellent and widely used tool. As we enter an age in which genome-wide studies are becoming increasingly frequent, the ability to efficiently simulate long chromosomal regions becomes more important. Consequently, the need for an efficient alternative to ms arises. While we feel that ms should continue to be the algorithm of choice when computational demands do not prohibit its use, we feel the SMC/SMC' algorithm provides a useful alternative in this new paradigm. In particular, it will lead to significant improvements in efficiency for methods such as rejection algorithms or computationally intensive simulation studies. While it models an approximation to the full coalescent model, the degree of approximation appears to be very good.

Software to implement the SMC algorithm is available as open-source, freely distributable C++ code. It can be used to generate data according to both the SMC and SMC' versions of the algorithm. The current implementation includes the possibility of allowing for changes in population size, in the form of an exponential growth model. This is dealt with in the standard way by altering the rates at which sequences coalesce (see [20], for example, for details). Clearly, there are a range of other complicating factors that one might wish to add to this code. Our view is that variation in recombination or mutation rates is best handled via post-processing the data produced by the standard form of the algorithm. For example, if one generates data for given values of *θ *and *ρ *one can produce recombination hotspots by contracting a region by a factor of *λ *(where *λ *is the relative rate of recombination in the hotspot) followed by a consequent thinning of mutations (including each mutation in that region with probability 1/*λ *if one wishes to keep the mutation rate constant). See [21] for details. The authors encourage interested parties to submit functions to allow for such complications. We will maintain these in a central repository.

## Conclusion

We have developed software *(FastCoal) *to implement the SMC algorithm of [6]. This algorithm approximates the standard coalescent process. We also introduce a modified version of the algorithm, SMC', which appears to produce a slightly closer approximation to the full coalescent model. The approximation makes the SMC/SMC' algorithm an appropriate choice for simulating long, chromosomal regions, for which existing algorithms become computationally intractable. We have shown that despite the fact that this method is an approximation to the exact coalescent model, it appears to produce data this is virtually indistinguishable from the exact model, at least in terms of patterns of pairwise LD and marginal TMRCAs. The behavior of LD is particularly relevant for genome-wide mapping studies, so we feel our results give convincing evidence that this software can be used to provide test data in a highly efficient manner when testing new genome-wide mapping methodologies.

## Availability and requirements

The *FastCoal *software, written in C++, is available from PM at pmarjora@usc.edu and runs on Windows platforms.

## List Of abbreviations

ARG – ancestral recombination graph;

LD – linkage disequilibrium;

MAF – minor allele frequency;

(T)MRCA – (time to) most recent common ancestor.

## Authors' contributions

PM and JDW are responsible for development and testing of the methodology. PM wrote the paper and C++ code.
